# Assessing the Alignment of Chilean Food-Based Dietary Guidelines on Cancer Prevention: A Content Analysis

**DOI:** 10.3390/healthcare13101133

**Published:** 2025-05-13

**Authors:** Alejandra Ortega-Guzmán, Solange Parra-Soto, María Jesús Vega-Salas, Lorena Rodríguez-Osiac, Sandra López-Arana

**Affiliations:** 1 Centro para la Transversalización de Género en I+D+i+e, Vicerrectoría de Investigación y Doctorados, Universidad Autónoma de Chile, Santiago 7500912, Chile; alejandra.ortega.g@cloud.uautonoma.cl; 2 Department of Nutrition and Public Health, Faculty of Health and Food Science, Universidad del Bío-Bío, Chillan 4051381, Chile; sparra@ubiobio.cl; 3 Center for Cancer Prevention and Control (CECAN), Santiago 7820436, Chile; muvega@uc.cl (M.J.V.-S.); lorenarodriguez@uchile.cl (L.R.-O.); 4 Departamento de Nutrición y Dietética, Escuela de Ciencias de la Salud, Facultad de Medicina, Pontificia Universidad Católica de Chile, Santiago 7820436, Chile; 5 Escuela de Salud Pública Dr. Salvador Allende, Universidad de Chile, Santiago 8380453, Chile; 6 Escuela de Nutrición y Dietética, Facultad de Medicina, Universidad Finis Terrae, Providencia 7501014, Chile

**Keywords:** cancer, Food-Based Dietary Guidelines, prevention, Chile

## Abstract

**Background/Objectives**: Cancer is a multifactorial disease. Evidence suggests that 30% to 50% of cancer incidence is linked to unhealthy behaviors. It is therefore important that dietary recommendations, including population-based dietary guidelines, and public policies be designed to support and facilitate healthier choices. We evaluated the alignment between the updated Chilean Food-Based Dietary Guidelines (FBDGs) and cancer prevention recommendations from the World Cancer Research Fund (WCRF/AICR) and the Latin America and the Caribbean Code Against Cancer (LAC-Code). **Methods**: A qualitative content analysis was performed based on the dissemination and technical documents from the updated Chilean FBDGs (2023), the WCRF/AICR and the LAC Code. A first set of codes was developed to evaluate the relevance of the messages with cancer prevention recommendations, and a second one aimed to characterize the food and diet recommendations included in cancer prevention recommendations and the Chilean FBDGs. Furthermore, we compared the 10 Chilean FBDG messages with the WCRF/AICR and the LAC Code recommendations. **Results**: The updated Chilean FBDG messages met most of the categories included. Four out of ten of the Chilean FBDG messages were directly aligned with LAC Code and the WCRF/AICR recommendations, five had an indirect relationship, and one was not related to any cancer recommendation. **Conclusions**: Our study suggests that the updated Chilean FBDG messages are most often indirectly aligned with the worldwide and the Latin American region cancer prevention recommendations over the role of diet, nutrition and physical activity in cancer risk among the general population.

## 1. Introduction

Cancer represents the second leading cause of death worldwide [1]. According to the World Health Organization (WHO), in 2022, there were an estimated 20 million new cancer cases and 9.7 million deaths [2]. In addition, it is expected that new cases will rapidly increase to 35 million by 2050 [3]. While in Latin America and the Caribbean region, cancer has become the leading cause of death in nearly half of all countries [4], Chile is no exception to this trend. For 2022, estimations of annual cancer incidence reached 59,876 cases and 31,440 deaths, with adjusted incidence rates of 140 and 103 per 100,000 male and female inhabitants, respectively [5]. This concerning scenario is characterized by population aging, and high prevalence of risk factors such as tobacco, alcohol, obesity, sedentary behavior and unhealthy diet, increasing the cancer burden to unreached levels [2].

Cancer is a multifactorial disease, which involves an array of genetic, medical, environmental and health-related behaviors [6]. Some evidence concludes that between 30% to 50% of cancer incidence is related to unhealthy behaviors [7]. Research has shown that between 5% and 30% of can be attributable to unhealthy dietary patterns [8]. Worldwide, it is estimated that 374,000 cancer deaths per year can be attributed to low fruit and vegetable intake [9,10,11]. It is therefore important that dietary recommendations, including population-based dietary guidelines, and public policies be designed to support and facilitate healthier choices.

As a first attempt, The World Cancer Research Fund (WCRF) and the American Institute for Cancer Research (AICR) have published three WCRF/AICR Expert Reports since 1997 and subsequently in 2007 and 2018 aimed to analyze and summarize the latest research worldwide on how diet, nutrition and physical activity affect cancer risk and survival [12]. The Third Expert Report translates its findings in 10 global cancer prevention recommendations [13]. Recently, the first edition of Latin America and the Caribbean Code Against Cancer (LAC-Code) was released [14]. This is an initiative to provide guidance to both the general population and policymakers regarding strategies to mitigate cancer risk. Recommendations include minimizing exposure to carcinogenic agents, adopting healthier behaviors, and engaging in screening tests with proven effectiveness [14].

On the other hand, the Food and Agriculture Organization of the United Nations (FAO) has prioritized the development of country specific Food-Based Dietary Guidelines (FBDGs) as which was convened in the International Conference on Nutrition in Rome in 1992 [15]. FBDGs are the preferred educational tool that provide information to the population to support and facilitate healthier choices. Since its implementation, the FBDG messages have shifted their focus from preventing undernutrition and micronutrient deficiencies to preventing obesity and non-communicable diseases (NCDs) as well as environmental sustainability [16]. This reflects the rapid nutritional transition in most countries, regardless of their development status, characterized by a steady increase in overweight and obesity prevalences [17].

To date, Chile has promulgated four FBDGs. The first FBDGs were published in 1997 and were known as “Food guidelines for the Chilean population”. Afterward, the Chilean FBDGs have been revised and updated in 2005, 2013 and 2022, respectively [18]. Despite the importance of following the FBDGs, more than two fifths Chilean adults did not meet the 2013 FBDGs, and evidence suggests that certain populations may find FBDGs especially difficult to comply with [19], and the effectiveness of these messages has not been extensively studied yet.

In the 2022 Chilean FBDGs, a series of messages was formulated and subsequently evaluated by experts and the general population to assess their relevance, importance and clarity. This evaluation aimed to bridge the gap between theoretical frameworks and practical application. A set of 10 messages was established, designed to guide the population toward adopting a healthier diet while respecting cultural contexts and promoting environmental sustainability. This new version did not include a specific message regarding physical activity [18].

In addition, no study has assessed whether the current Chilean FBDGs comply with the cancer prevention recommendations included in WCRF/AICR (2018) [13] and in the LAC-Code [20]. Therefore, this study aimed to evaluate the alignment between the updated Chilean Food-Based Dietary Guidelines (FBDGs) and cancer prevention recommendations from WCRF/AICR and LAC-Code using a qualitative content analysis. This analysis will provide essential insights into the strengths and weaknesses of the current Chilean FBDGs and serve as a tool to develop more effective strategies for their dissemination and implementation, ultimately leading to improved health outcomes for the Chilean population.

## 2. Materials and Methods

**Figure 1:**
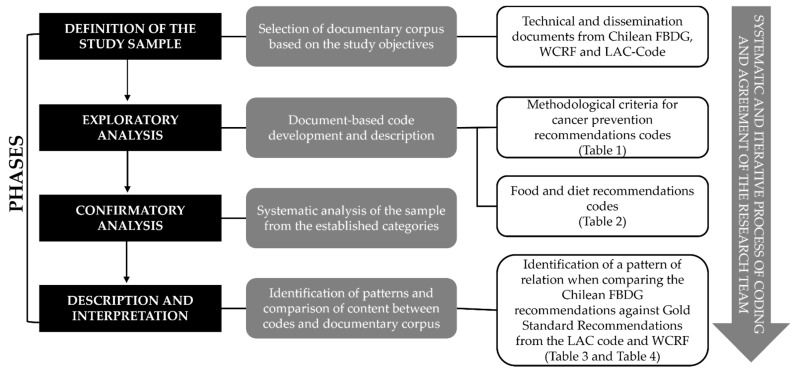
Methodological flowchart based on the information processing model published by Flores (2009) [21].

This qualitative study used the information processing model published by Flores (2009) [21], which schematically shows the phases usually considered in qualitative data analysis. In Figure 1, we present the flowchart that shows the four phases followed in the present study and that we detail below.

(1) **Definition of the study sample:** to evaluate the alignment of the 2023 Chilean FBDG messages to the gold standard cancer prevention recommendations, we included the documents from the WCRF/AICR (2018) and the LAC-Code (2023) because they include up-to-date evidence worldwide and from the Latin American region on how diet, nutrition and physical activity influences cancer risk in the general population. We analyzed the technical and dissemination documents of the Chilean FBDGs [22,23], the WCRF/AICR [12,13], and the LAC-Code [14,20] in order to gain a comprehensive understanding of the criteria and final messages, totaling five documents.
(2) **Exploratory analysis**: based on the qualitative content analysis [21] and the iterative and open approach to the data suggested by the Grounded Theory [24], we conducted a first approach to the documentary corpus to identify the main criteria used to build the cancer prevention recommendations according to the gold standard documents [13,14] and the main topics addressed in all dissemination documents [13,20]. This phase resulted in a tree code with eight preliminary categories to analyze the Chilean FBDGs in terms of their content and approach to cancer prevention (Table 1). A second tree code was built with fifteen preliminary categories to analyze the ten Chilean FDGB messages with topics related to food and diet identified in both cancer-related technical reports (Table 2).

(3) **Confirmatory analysis:** considering qualitative content analysis as a technique that necessitates an objective and systematic description of the categories or codes to be used [21], we proceeded to describe and define all the categories, eventually reaching a consensus among the researchers. Once we agreed on how to comprehend and apply each code, the researchers examined the sample independently. The iterative process was conducted as a dyad of independent coding and collective reflection on the findings in monthly meetings until all the documents were analyzed. In these meetings, the research team met to share their results and reflect on them, discussing the meaning of the findings and the themes that appeared relevant in the documents, reaching a consensus in their interpretation considering the coding criteria, the content of the documents and their expert knowledge on the theme.
(4) **Description and interpretation**: to integrate the results from the coded documents, we conducted a thorough analysis of the results, identifying patterns and comparing the information across categories, keeping the iterative process of independent coding and collective reflection as described in stage 3. This resulted in the recommendations to include cancer prevention in the Chilean FDGB in the future, based on the tree code described in Table 1, as well as an analysis of a thematic relationship between the updated Chilean FBDGs and the key topics identified as relevant for cancer prevention from a dietary perspective, using the findings from the tree code described in Table 2. In this case, three potential links were established between the topics identified: direct, indirect, and null, depending on how both the content and approach were addressed in the gold standard documents and the Chilean FBDGs. All three categories are described in Table 3. All tree codes were used to analyze the 10 messages of the updated Chilean FBDGs.

The iterative and precise process for the construction of codes and subsequent analyses was held as a strategy to maintain the criteria of reliability and rigor in research, a relevant element to ensure the quality of qualitative research [25]. This structured and agreed-upon framework allowed us to conduct a comparative analysis with common criteria, evaluating the congruence between national guidelines and international standards on cancer prevention and nutrition.

## 3. Results

This analysis related to content and approach to cancer prevention in the Chilean FBDGs yielded the following findings:

The Chilean FBDGs complied with the ***scientific-evidence-based*** criterion established by the gold standard reference. The disclosure report (FBDG educational handbook) shows a range of scientific assertions concerning food properties, including its composition, absorption mechanisms, and effects on metabolism. Likewise, it addresses the implications of food-related practices, such as greenhouse emissions and plastic usage, as well as the advantages associated with specific food behaviors, including eating with friends and family, home cooking, and the reduction in food waste. Nonetheless, these assertions are not explicitly attributed to scientific evidence, due to the nature of the disclosure report. To address this issue, we performed a search for supporting evidence in the technical document. Most of these statements were appropriately backed by relevant scientific references, or they dealt with topics already studied and verified in the previous Chilean FBDGs, such as food safety recommendations or those related to the nutritional content of certain foods such as legumes, fish and shellfish, thus fulfill the criterion of being supported by scientific evidence.

One of the unique topics included in the new Chilean FBDGs was the ***local context.*** The disclosure report emphasizes the environmental and lifestyle conditions within the country and its impact on dietary behaviors. This local context is characterized by promoting unhealthy habits, evidenced by the “*excessive intake of energy, fats, and sugars that disrupts energy balance and make us sick slowly but progressively*” by diminishing opportunities to access to local growing food and fostering sedentary activities due to urbanization and technological development. Specifically, the updated Chilean FBDGs consider this local context by examining the ecological consequences of food system practices, the health-related behaviors, and issues surrounding food availability. The guidelines provide explicit recommendations that encourage the selection of locally sourced fresh foods from markets and open-air markets, promote the preservation and transmission of culinary knowledge, and encourage accessible food across the country. Collectively, these strategies aim to enhance population health and mitigate adverse environmental impacts.

In the technical document, this approach is reflected in the explicit inclusion of socioeconomic and cultural variables in the bibliographic searches of all the technical chapters included, in addition to the question of characterizing local food cultures within the country and its corresponding correlation with the methodology and the construction of a sample to validate the new guidelines in all macro-zones of the country. Nonetheless, the component of “*addressing a particular risk factor or an effective intervention in terms of regional cancer burden*” is not mentioned to address contextual or environmental factors influencing cancer incidence. Instead, it mainly focuses on individual behaviors related to the promotion or avoidance of specific dietary choices, emphasizing personal health outcomes and the reduction in “excess fat deposits”. This perspective neglects structural interventions and the examination of detrimental environmental impacts.

The updated Chilean FBDGs indicate its ***population-based*** nature. It does not focus on a particular demographic group, although it targets those over 2 years of age. At the same time, it states that it addresses biological, nutritional, social and cultural dimensions. This approach is also stated in the epidemiological characterization at the technical document, included in the chapters specifically aimed at addressing the effects of food over population health. However, some topics are specifically addressed to pregnant women, children, older people, and people with dietary restrictions such as vegetarians and vegans, as special population groups. For example, emphasis is placed on valuing the knowledge of traditional recipes that older people probably have. Specific recommendations are also made for boys and girls regarding the inclusion of cooking tasks, encouraging iron consumption to support their growth, and eating in company as a protective effect against NCDs. Concerning pregnant women, the importance of increasing dairy consumption is mentioned, and among vegetarians or vegans, consultation with a healthcare professional is recommended. Like the *local context* code, there is no specific focus on cancer from a dissemination perspective in the disclosure document, despite addressing the prevalence of cancer in both the general population and specific groups in the technical document.

The Chilean FBDGs are an important public policy tool, and some recommendations stressed the relevance of ***public policy endorsement*** to comply with them. In the dissemination document, the authors explicitly outline in its formulation and scope that strong social participation in the guide’s elaboration process is needed to address the current challenges of the local context including stakeholders and policymakers. It also links some recommendations with other public policies such as the recommendation on dairy consumption and the provision of nutritious dairy products through the National Complementary Feeding Program (PNAC by its Spanish acronym) and the Complementary Feeding Program for the Elderly (PACAM by its Spanish acronym) targeted to the most disadvantage population. Similarly, the recommendation to reduce the consumption of ultra-processed foods and those high in energy and nutrients of concern, identified by the front-of-package warning labels implemented by Law 21.606 on Food Labeling and Advertising. This aligns with the recommendations made by the panel of experts in the technical document, who emphasize the importance of making comprehensive public policy decisions. The connection of these policies with cancer prevention is not explicitly presented, but rather only in relation to other NCDs and the prevention of obesity, diabetes, and cardiovascular diseases.

In the updated Chilean FBDGs, the creation of new messages tried to “*minimize the gaps between theory and practice*” as well as to guide the population using graphic representations, simple and assertive language, and the incorporation of practical examples to facilitate the implementation of the 10 messages, following the recommendation for ***clear communication:*** In the methodological aspects of the technical document, the development and validation of messages for the population and an attractive graphic proposal are described as fundamental dimensions, which were finally reflected in the dissemination document. Despite the above, and as occurs in the previous criteria, the link with cancer prevention is not explicit.

Regarding the ***dietary pattern approach,*** the new Chilean FBDGs maintain some recommendations associated with isolated foods or food groups, such as recommendations for the consumption of legumes, fruits, vegetables, fish, seafood and dairy products. Each chapter in the dissemination document includes information in which these foods are contextualized within everyday scenarios, such as recipes to cook meals or snacks. It also shows examples of integrating the foods or food groups into preparations as part of a healthy diet. Similarly, in the technical document, the idea of regularly incorporating a variety of natural foods into the diet is reinforced, avoiding the use of supplements, and as a preventive strategy for various NCDs.

The link to cancer prevention in the disclosure document is made based on singular types of foods, associating the properties of fruits and vegetables with the prevention of some types of cancer, and highlighting that frequent consumption of ultra-processed foods is associated with an increased risk of cancer. The technical document incorporates strong evidence linking diet and cancer prevention, emphasizing the benefits of calcium supplementation, a healthy dietary pattern rich in fruits, vegetables, low-fat foods, fish, poultry and legumes against colorectal cancer, and the role of breastfeeding in reducing the risk of certain types of cancer.

The recommendations were ***realistic and achievable*** as they included an explicit connection to the national cultural context and the safeguarding of traditional practices, as fundamental components of the updated Chilean FBDGs. It is also mentioned in the development of the messages and specifically claimed in message number eight: “*Share the kitchen tasks, enjoying new and traditional preparations*”. Similarly, in the disclosure report, there is a chapter that provides a comprehensive framework for integrating the FBDG messages into daily routines, while also presenting a recipe collection that showcases diverse culinary preparations representative of the country’s variety of food cultures. This approach is closely related to the “*dietary pattern approach*”, which also contributes to placing food in relation to its environment and social use, as well as to the sociocultural practices associated with the supply, preparation and consumption of food (commensality, culinary practices, etc.).

In the technical report, the realistic and achievable criterion is shown in the scientific review that considers the sociocultural dimensions of food that are pertinent to examine within a national context. This includes the symbolic meanings attributed to food, the practices individuals and communities engage in throughout the food system—encompassing food acquisition, processing, consumption, and disposal—and the overarching cultural frameworks surrounding food. Despite the above, the link with cancer prevention is not explicit.

The foundational principle of the new Chilean FBDGs emphasizes the promotion of healthy dietary practices as a ***preventative measure against various diseases***. The primary focus is directed towards NCDs, specifically obesity, diabetes, and cardiovascular diseases, with cancer and other neurological disorders receiving secondary consideration. In the disclosure document, references to cancer are less prominent in comparison to other NCDs. Cancer is explicitly addressed primarily in connection with established links between dietary components and their potential role as either protective factors or promoters of the disease, as elaborated in the *dietary pattern approach dimension*. Cancer is explicitly referenced in the message: “*Add color and flavor to your day with vegetables and fruits in everything you eat*”, highlighting its potential for prevention through the consumption of fruits and vegetables, particularly those endowed with antioxidant and anticancer properties, such as purple, red, and green varieties. Similarly, the recommendation to “Eat legumes in stews and salads as often as you can” indicates that legumes may serve as protective foods against cancer and other NCDs. Additionally, the guideline to “*Avoid ultra-processed products and those with ‘HIGH IN’ labels*” suggests an association between the consumption of such labeled products and the increased risk of developing cancer and other NCDs.

Conversely, the technical document provides substantial evidence related to cancer. This includes findings from an epidemiological study conducted to support the current guidelines, indicating that cancer has emerged as the leading cause of death within the Chilean population. Furthermore, the document addresses the influence of specific food items and dietary patterns on the prevention and incidence of specific cancers. Nevertheless, it is noteworthy that not all the evidence is directly associated with the recommendations, particularly concerning the potential of nutritional factors, as articulated in the guidelines, to mitigate the risk of this disease.

Related to the content match, the results of the comparative descriptive analysis between the updated Chilean FBDG messages and the recommendations from the WCRF/AICR and the LAC-Code are summarized in Table 4.

Among the total number of messages present in the Chilean FBDGs, four exhibit a direct relationship in their content with the recommendations outlined in the LAC Code and the WCRF/AICR documents. The messages “*Add color and flavor to your day with vegetables and fruit in everything you eat*” and “*Eat legumes in stews and salads as often as you can*” align closely with the recommendations outlines in the LAC Code, which indicates “*Eat as many vegetables and fruits as possible at each meal, and regularly include legumes such as beans and lentils*”, and of the WCRF/AICR, which recommends “*Eat a diet rich in wholegrains, vegetables, fruit and beans*”. In all three documents, they show strong evidence linking the consumption of these foods and the prevention of various types of cancer. They emphasize the importance of a diverse diet rich in fiber and nutrients, which significantly contributes to the maintenance of overall health. Water consumption is another topic with a direct relationship between the documents analyzed. The Chilean guidelines advise to “*Drink water several times a day; do not replace it with juices or soft drinks*”. Similarly, the LAC Code underscores the message to “*Avoid sugar-sweetened beverages, drink water instead*”, while the WCRF/AICR recommends the following: “*Limit consumption of sugar sweetened drinks*”. All documents highlight the health benefits of drinking water in terms of hydration, but also because it is the best substitute for other sugary drinks that are related to weight gain, obesity, and a risk factor for the appearance of cancer. Despite agreeing on the importance of promoting its consumption, the Chilean guidelines do not explicitly state its link with cancer prevention, despite establishing such associations for other NCDs.

The recommendation to avoid ultra-processed foods is also directly related to the documents reviewed. In the Chilean FBDGs, it is stated to “*Avoid ultra-processed products and those with “HIGH IN” labels*”. Likewise, in the LAC Code it is expressed as follows: “*Limit your consumption of ultra-processed foods, such as sweets, sweetened breakfast cereals, salty snacks, pastries, and cookies, among others. Instead, eat natural foods or foods prepared at home*”. Furthermore, the WCRF/IARC suggests to “*Limit the consumption of “fast foods” and other processed foods high in fat, starches or sugars*”. In this sense, all the documents clearly emphasize avoiding the consumption of ultra-processed foods, linking them to weight gain, NCDs and cancer. The gold standard documents advocate for the implementation of public policies that promote healthier food environments, such as fiscal measures and warning labels. Notably, such policies have been enacted in Chile and align with the recommendations set forth in the WCRF/IARC and LAC Code recommendations. This is an example of the intersection between cancer prevention strategies and prevailing public policy initiatives within the local context.

In five recommendations of the Chilean FBDGs, an ***indirect relationship*** is observed with the contents established in the LAC Code and the WCRF/IARC. The Chilean FBDG recommendations emphasize the acquisition of fresh food in established markets, and it is explicitly recommended in the following messages: “*Eat fresh food from established Open-Air Markets and markets*” and “*Increase consumption of fish, shellfish or seaweed from authorized places*”. This approach aims to enhance short supply chains, promote seasonality, and bolster sustainability. In particular, the recommendations for fish and seafood consumption highlight the importance of obtaining these products fresh and ensuring proper handling to prevent contamination and the transmission of foodborne illnesses.

Comparatively, the gold standard documents do not offer direct equivalents to these specific recommendations. Instead, the LAC Code and the WCRF/IARC emphasize the importance of building healthy environments where such food options are accessible. Notably, the consumption of fish and seafood is not specifically addressed within the LAC Code; however, the WCRF/IARC acknowledges its presence in dietary guidelines across various nations, advocating for fish and seafood consumption as a substitute for red meat. In the WCRF/IARC report, the panel of experts described the possible association between the culinary preparation of Cantonese-style salted fish and nasopharyngeal cancer, discouraging its consumption for both adults and children. In contrast, the Chilean FBDGs did not explicitly establish the relationship between fish consumption and cancer. Fish is characterized as a protective factor against other NCDs, emphasizing its contribution of high-quality proteins and essential nutrients, including Omega-3 fatty acids.

As previously mentioned, the Chilean FBDGs incorporate recommendations concerning the sociocultural dimensions of food. These guidelines emphasize the significance of contextualizing food within its preparation and consumption environments, the transmission of culinary practices that embody cultural traditions, and the social interactions promoted through communal food sharing. In this sense, the messages “*Share kitchen tasks, enjoying new and traditional preparations*” and “*Enjoy your food at the table, eat with others when you can, and take away the screens*” highlight these aspects and relate them to the benefits of putting them into practice to improve quality of life and health outcomes.

Although there is no explicit link to cancer prevention, these topics are partially included in the WCRF/IARC recommendations, specifically in “*Eat a diet rich in wholegrains, vegetables, fruit and beans*” to highlight the importance of considering local practices and traditional food systems when recommending the consumption of fruits and vegetables. This also represents a challenge for public policies to create initiatives that are sufficiently comprehensive to address such local contexts. Similarly, the WCRF/IARC in its recommendation “*Be physically active*” highlights that screen time is associated with a sedentary lifestyle and higher intake of ultra-processed foods, advocating for its reduction, while it does not address commensality, unlike the Chilean FBDGs. On the other hand, the LAC Code does not specifically address these issues; however, it emphasizes the significance of food environments that promote healthy practices among the population. In this context, one could infer a potential reduction in screen time and an encouragement for increased shared meals.

Specifically, regarding sustainability, the Chilean FBDGs recommend the following: “*Protect the planet, take care of the water, do not throw away food, separate your trash and recycle*”. This topic does not establish an explicit relationship with cancer in the disclosure document. In the gold standard documents, the LAC Code proposes minimizing outdoor exposure during air pollution episodes and advises against the accumulation of indoor smoke when using fuels like charcoal or firewood for cooking. Conversely, for the WCRF/IARC, the concept of sustainability is mentioned in connection with both the direct and indirect impacts of food production on greenhouse gas emissions. Particular attention is directed towards the production of processed red meats, which exhibit a strong association with the incidence of colorectal cancer. Consequently, the WCRF advocates for the adoption of a plant-based dietary pattern to promote both human health and environmental sustainability.

Finally, in comparison to the reference documents from WCRF/IARC and LAC Code, the Chilean FBDGs on dairy consumption present a ***null relationship***. The Chilean FBDGs indicate the following: “*Consume dairy at all stages of life*”, emphasizing the presence and bioavailability of nutrients found in these products, which contribute to the prevention of non-communicable diseases such as obesity, cardiovascular diseases, and type 2 diabetes mellitus. They also highlight the potential benefits of dairy in preserving muscle mass and bone health. In contrast, the reference documents neither recommend nor discourage dairy consumption. Therefore, this topic is excluded from their recommendations. Instead, these documents prioritize the promotion of breastfeeding as a protective measure against breast cancer and excessive weight gain. Both referenced organizations urge stakeholders to implement strategies that facilitate breastfeeding practices in public and workplace settings, as well as to regulate the marketing of breast milk substitutes. Notably, this aspect is not addressed within the Chilean national guidelines.

## 4. Discussion

Cancer is a serious and growing problem in Chile [5]. Knowing that many types of cancer are preventable by changing behaviors [4], and that those changes require changes in the most structural determinants, it is relevant to have public policies that address these determinants [26]. FBDGs are making cancer prevention explicit among their objectives and aligning their messages with international recommendations and available scientific evidence.

In this study, we aimed to evaluate the alignment of the messages from the updated Chilean FBDGs with cancer prevention recommendations stated by the WCRF/AICR and the LAC Code. Overall, the messages met most of the criteria of being based on sufficient scientific evidence, locally context-based, population-focused, endorsing public policies, being clear and acknowledging the Chilean dietary pattern, achievable across different cultures and considering the prevention of other diseases. Nonetheless, the focus is not specifically on cancer prevention but rather on NCDs. Additionally, our findings highlight a mixed alignment between Chilean FBDGs and the WCRF/AICR and the LAC Code cancer prevention recommendations. Four Chilean FBDG messages displayed a direct relation, half demonstrated a more indirect relation, and one message had a null relation to the cancer prevention recommendations stated by the WCRF/AICR and the LAC Code.

The updated Chilean FBDGs are based on scientific evidence, which is very well reflected in the technical document of the guidelines, but the explicit link to cancer is not always indicated in the disclosure document. In recent years, some authors have called for more transparency on the process used to develop dietary guidelines [27,28,29]. Blake et al. analyzed 32 FBDGs, reporting that some inconsistencies remain in the methods used for evidence review, evaluating evidence quality, and grading recommendations [30]. In the development process for the 2020–2025 Dietary Guidelines in the United States, four phases were considered: (1) identifying the topics and supporting scientific questions to be examined, (2) appointing a Dietary Guidelines Advisory Committee to review current scientific evidence and develop a scientific report, (3) developing the new edition of the Dietary Guidelines, and (4) implementing the Dietary Guidelines through federal programs and nonfederal program entities [31]. All of these phases were introduced to avoid a lack of transparency in guideline committees, to thoroughly analyze quality of systematic reviews underpinning guidelines, and to provide more clarity about how evidence is finally translated into public health recommendations [28].

After comparing the messages from the updated Chilean FBDGs with the reference documents, 40% adequately describe a direct alignment to cancer prevention recommendations. These include adherence to fruit and vegetables, legumes and water consumption. It has been reported that consuming non-starchy vegetables and/or whole fruit “probably” protects against several aerodigestive cancers, including mouth, pharynx, larynx, nasopharynx, esophagus, lung, stomach and colorectal cancers [9,13,32]. In addition, it is also recognized that adhering to healthy diets has a lower environmental impact compared to the average current diet [16]. On the other hand, the comparative analysis found that the analyzed documents recommend limiting the consumption of ultra-processed foods high in saturated fat, starches, or added sugars. The health effects of ultra-processed foods have gained increased attention in public health discussions. Dietary guidelines have incorporated advice about ultra-processed foods following the Nova food classification system [33]. According to Koios et al., out of the 106 guidelines analyzed, 91 provided recommendations related to food processing. These recommendations might address ultra-processed foods in three ways: (1) by explicitly mentioning ‘ultra-processing’ or ‘ultra-processed foods’, (2) by advising against certain specific ultra-processed items, or (3) by highlighting characteristics that suggest the presence or absence of ultra-processing [34].

In the Chilean FBDG messages, water intake is recommended as the main drink, limiting the consumption of other drinks, particularly sugar-sweetened beverages. Water is essential for the survival of organisms, as it helps regulate temperature, safeguards tissues and organs, facilitates the absorption and transport of nutrients to cells, and supports various other critical bodily functions [35]. For that reason, all national-based documents established recommendations related to water consumption. Nonetheless, the number of servings per day in the updated Chilean FBDGs was not established, restricting the ability for the population to compare their intakes against a recommendation.

Additionally, our comparative analysis shows that the updated Chilean FBDGs did not include any recommendations about breastfeeding probably because the Chilean FBDGs are aimed at the population over 2 years of age. These results are similar those shown by Martini et al., who found that messages regarding breastfeeding and complementary feeding were included in only 56% of total 43 FBDGs analyzed worldwide [36]. Instead, the Chilean FBDGs do include a specific message for dairy consumption. In United States the American Cancer Society did not make a specific recommendation regarding dairy food consumption for cancer prevention because intakes of dairy foods may decrease the risk of some cancers and possibly increase the risk of others [32]. The Third Expert Report from the WCRF/AICR found evidence that calcium and dairy products increase prostate cancer risk. For every 400 g of dairy intake (equivalent to almost two cups of milk per day), prostate cancer risk was 11% higher [10,12]. The Chilean FBDGs recommend consuming at least three servings/day (±600 mL/day). In the Latin American region, the daily consumption of dairy products ranged from one to five servings/day, highlighting the large variation within this region [37]. On the contrary, the Eat Lancet commission recommends reducing dairy intake to 250 g/day, in an effort to reduce the environmental effects of dairy production and consequently promote global healthy and sustainable diets [38]. Future studies should seek to determine a balance between dairy consumption recommendations that combine both nutritional requirements and sustainability for reducing the environmental effects of dairy production systems.

Our results show there is room for improvement regarding cancer prevention. Half of the recommendations have an indirect relationship with the reference documents of cancer organizations, considering they refer to contextual variables for cancer prevention such as food environments and other environmental factors. These challenges are critical for public policy initiatives that address public health problems in general and all NCDs in particular, from a structural standpoint, where individual acts are viewed in a broader context. The background papers use this approach. Although the messages focus on individual action, they also provide recommendations for decision- and policymakers.

On the other hand, to highlight the potential of FBDGs as a cancer prevention tool, Brazil’s FBDGs [39] establish a more direct relationship between the recommendations and the prevention or occurrence of certain types of cancer and cite the WCRF/AICR study. A similar issue is that of Uruguay’s FBDGs [40], which repeatedly mention the guidelines’ association with cancer in addition to containing cautions regarding alcohol use, a topic not covered in the Chilean FBDGs.

Our study is the first to assess alignment between current Chilean dietary guidelines and international cancer prevention recommendations using a comprehensive and contextualized content analysis. This approach yields an extensive perspective on both the alignment and discrepancies between Chilean dietary recommendations and those established internationally.

Nonetheless, the study is not without limitations, which should be acknowledged and addressed in future research endeavors to enhance the generalizability and applicability of the findings. The qualitative perspective of the analysis may impose constraints on the broader applicability of the results. While the analysis was conducted under a rigorous and systematic methodology, the inherent subjectivity associated with qualitative content analysis may influence both the interpretation of the data and the resulting conclusions. Additionally, it is important to recognize that information and recommendations are subject to change, as new scientific evidence emerges. Consequently, the findings of this study should be periodically reviewed and updated to maintain their relevance and accuracy within the evolving landscape of dietary guidelines and cancer prevention strategies. Further FBDGs in Chile and other contexts should actively incorporate international cancer prevention recommendations when developing population-based dietary recommendations and promote healthier and sustainable diets for all.

## 5. Conclusions

This study assessed the alignment of the current Chilean FBDGs with international cancer prevention recommendations. Our findings suggest that the Chilean FBDG messages exhibit an indirect alignment with evidence on the relationships between diet, nutrition, physical activity, and cancer risk in the general population. Recognizing that FBDGs play a crucial role as communication tools for both the public and policymakers, these results underscore the need for future updates to explicitly integrate cancer prevention rationales and evidence-based messages. This includes ensuring that dietary recommendations explicitly reflect both international and national evidence linking specific dietary patterns to reduced cancer risk. Furthermore, strengthening intersectoral collaboration in the dissemination and implementation of the guidelines is essential to maximize their reach and adoption. It is critical to establish systems for monitoring and evaluating the implementation and effectiveness of the FBDGs, with a focus on their impact on reducing cancer in Chile. Lastly, as a future research perspective, it is important to emphasize that physical activity offers numerous health benefits and significantly contributes to the well-being and prognosis of cancer survivors. Efforts are needed to ensure equitable access to safe and supportive environments that promote both healthy eating and regular physical activity. This comprehensive approach aims to improve individual and community health while advancing social, cultural, and economic development.

## Figures and Tables

**Table 1:** Set of methodological criteria for cancer prevention recommendations.

Criterion	Definition	Food-Based Dietary Guidelines Application
Scientific evidence-based	“The recommendation should be based on sufficient scientific evidence that following the recommendation to avoid or reduce exposure to a harmful agent, to adopt a healthy behavior, or to uptake a medical intervention, would reduce the individual’s risk of developing cancer or dying from cancer” [14].	Explicit reference of the scientific sources that support the information provided and its relationship with cancer prevention.
Local context	The recommendation should be relevant to address a “particular risk factor or an effective intervention in terms of regional cancer burden”, including “the social conditions and health inequalities in the region, as well as the availability, feasibility, and affordability of the proposed interventions according to the LAC settings” [14].	Identification of the sociodemographic characteristics of the Chilean context, and their incidence in the availability, feasibility, and affordability of the recommendations and its relationship with cancer prevention.
Population focused	“The recommendation should target large segments of the general population of the whole region or sufficiently large subregions (i.e., not high-risk groups or special small subpopulations), so that it is relevant to have the whole general population informed” [13,14].	Identification of the subjects of the FBDGs and whether they target specific population groups, and its relationship with cancer prevention.
Public Policy endorsement	The recommendation should be endorsed by public policies that enable it to comply, “so that individuals do not have to take autonomous decisions” and also “to further support guidance for policymaking or for policy changes when possible” [14].	Explicit mention of current Chilean public policies that support the implementation of the action recommended and its relationship with cancer prevention.Structural-focused vs. individual-focused recommendations and its relationship with cancer prevention.
Clear communication	The recommendation should be presented as a clear message “to achieve uptake of the recommendations by the public” [14].	Explicit consideration of actions to deliver a clear message in terms of language use, graphic resources and additional information to support the FBDGs.Final FBDG document analysis of technical language use, graphic resources to support the recommendation, and additional information and how it complements and makes the implementation easier for the public, and its relationship with cancer prevention.
Dietary pattern approach	The recommendation should consider the diet or eating patterns as a whole, focusing on the interaction of foods, drinks, and nutrients, because it is hard to identify “singular effects attributable to individual dietary or other factors” [13].	Focus on the diet as a whole vs. the food isolated from its use and context, and its relationship with cancer prevention.
Realistic and achievable	The recommendation should be culturally relevant for the population, emphasizing “aspects of diet and nutrition, physical activity and body fatness that protect against cancer and can be achieved across different cultures” [13].	Mention of cultural context in the formulation of the FBDGs, using examples of regional foods and preparations and its relationship with cancer prevention.
Prevention of other diseases	The recommendation should “take into account the prevention of other diseases besides cancer” [13].	Explicit mention of diseases in general and cancer in particular into the guidelines (frequency and association).

**Table 2:** Set of food and diet recommendations included in cancer prevention recommendations and Chilean FBDGs.

Criterion	Definition
Fresh food acquisition	It refers to the purchase of food in its natural form and the supply places where to acquire them.
Fruit and vegetable consumption	It refers to the intake recommendation of fruits and vegetables as a food group in general or to any food that can be classified within it. It includes preparations that can use them as ingredients.
Legume consumption	It refers to the intake recommendation of legumes as a food group in general or to any food that can be classified within it. It includes preparations that can use them as ingredients.
Whole grain consumption	It refers to the intake recommendation of whole grains as a food group in general or to any food that can be classified within it. It includes preparations that can use them as ingredients.
Water consumption	It refers to the intake recommendation of water in any context in day-to-day life. It includes preparations where it can be used as an ingredient.
Dairy consumption	It refers to the intake recommendation of dairy products as a food group in general or to any food that can be classified within it. It includes preparations that can use them as ingredients.
Red meat consumption	It refers to the intake recommendation of red meat as a food group in general or to any food that can be classified within it. It includes preparations that can use them as ingredients.
Alcohol consumption	It refers to the intake recommendation of alcohol in general or to any product that can be classified as alcohol.
Sea food consumption	It refers to the intake recommendation of sea food as a food group in general or to any food that can be classified within it. It includes preparations that can use them as ingredients.
Ultra-processed foods consumption	It refers to the intake recommendation of ultra-processed foods as a food group in general or to any food that can be classified within it. It includes preparations that can use them as ingredients.
Dietary supplements	It refers to the intake recommendation of dietary supplements in general, including any population group or health condition.
Breastfeeding	It refers to the mention of any recommendation related to breastfeeding.
Sociocultural dimension of food	It refers to the consideration of any contextual aspect where food consumption takes place, including recommendations for culinary techniques, culturally relevant recipes, communal dining, and roles, among others.
Environmental care	It refers to the consideration of any aspect related to the environmental care of food environments. Including mentions of food production methods, waste management, supply chains, and the impacts of these practices on the environment.
Physical Activity	It refers to recommendations related to body movement in any context of human life (occupational, sports, transportation, etc.)

**Table 3:** Possible relationships between food and dietary topics addressed in cancer prevention recommendations and Chilean FBDGs.

Criterion	Definition
Direct	The Chilean FBDGs included the same content and approach of the gold standard recommendations, without contradictions or missing topics.
Indirect	The Chilean FBDGs included some topics but were not addressed exactly as the gold standard recommendations.
Null	The Chilean FBDGs did not address any of the recommendations or the topics included in the gold standard recommendations.

**Table 4:** Set of criteria for qualitative comparative analysis between the Chilean FBDG messages and the LAC Code and the WCRF/AICR for nutrition and diet recommendations.

Chilean FBDG Message	LAC Code Recommendation	WCRF/AICR Recommendation	Relationship
Eat fresh food from established Open-Air-Markets and markets	N/A	Eat a diet rich in wholegrains, vegetables, fruit and beans	Indirect
Add color and flavor to your day with vegetables and fruits in everything you eat	Eat as many fruits and vegetables as possible at each meal, and regularly include legumes such as beans and lentils	Eat a diet rich in wholegrains, vegetables, fruit and beansDo not use supplements for cancer prevention	Direct
Eat legumes in stews and salads as often as you can	Eat as many fruits and vegetables as possible at each meal, and regularly include legumes such as beans and lentils	Eat a diet rich in wholegrains, vegetables, fruit and beans	Direct
Drink water several times a day, do not replace it with juices or soft drinks	Avoid sugar-sweetened beverages, drink water instead	Limit consumption of sugar sweetened drinks	Direct
Consume dairy at all stages of life	Breastfed your baby—the more months the better—to help prevent breast cancer and excess weight in your baby	For mothers: breastfeed your baby, if you can	Null
Increase consumption of fish, shellfish or seaweed from authorized places	N/A	N/A	Indirect
Avoid ultra-processed products and those with “HIGH IN” labels.	Limit your consumption of ultra-processed foods, such as sweets, sweetened breakfast cereals, salty snacks, pastries, and cookies, among others. Instead, eat natural foods or foods prepared at home	Limit consumption of “fast foods” and other processed foods high in fat, starches or sugars	Direct
Share the kitchen tasks, enjoying new and traditional preparations	N/A	Eat a diet rich in wholegrains, vegetables, fruit and beans	Indirect
Enjoy your food at the table, eat with others when you can, and take away the screens	N/A	Be physically active	Indirect
Protect the planet, take care of the water, do not throw away food, separate your trash and recycle	N/A	N/A	Indirect

## Data Availability

All documents from the Chilean Food-Based Dietary Guidelines were obtained through the publicly available contents on https://www.minsal.cl/guias-alimentarias-para-chile/ (accessed on 19 November 2024).

## Abbreviations

The following abbreviations are used in this manuscript:

FBDG	Food-Based Dietary Guidelines
WCRF/AICR	The World Cancer Research Fund and the American Institute for Cancer Research
LAC-Code	Latin America and the Caribbean Code Against Cancer
